# Self-reported disability in rural Malawi: prevalence, incidence, and relationship to chronic conditions

**DOI:** 10.12688/wellcomeopenres.15196.4

**Published:** 2020-12-22

**Authors:** Josephine E. Prynn, Albert Dube, Elenaus Mwaiyeghele, Oddie Mwiba, Steffen Geis, Olivier Koole, Moffat Nyirenda, Hannah Kuper, Amelia C. Crampin

**Affiliations:** 1 Malawi Epidemiology and Intervention Research Unit (MEIRU), Lilongwe, Malawi; 2 University College London, London, UK; 3 London School of Hygiene & Tropical Medicine, London, UK; 4 Institute for Medical Microbiology and Hygiene, Phillips University of Marburg, Marburg, Germany; 5 MRC/UVRI Uganda Research Unit, Entebbe, Uganda

**Keywords:** Disability, Prevalence, Malawi, Africa, Chronic disease, Non-communicable disease, HIV, Health and Demographic Surveillance Site

## Abstract

**Background:** Disability is a complex concept involving physical impairment, activity limitation, and participation restriction. The Washington Group developed a set of questions on six functional domains (seeing, hearing, walking, remembering, self-care, and communicating) to allow collection of comparable data on disability. We aimed to improve understanding of prevalence and correlates of disability in this low-income setting in Malawi.

**Methods:** This study is nested in the Karonga Health and Demographic Surveillance Site in Malawi; the Washington Group questions were added to the annual survey in 2014. We used cross-sectional data from the 2014 survey to estimate the current prevalence of disability and examine associations of disability with certain chronic conditions. We then reviewed the incidence and resolution of disability over time using panel data from the 2015 survey.

**Results:** Of 10,863 participants, 9.6% (95% CI 9.0-10.1%) reported disability in at least one domain. Prevalence was higher among women and increased with age. Diabetes and obesity were associated with disability among women, and diabetes was also associated with disability among men. Neither hypertension nor HIV were associated with disability. Participants reporting “no difficulty” or “can’t do at all” for any domain were likely to report the same status one year later, whereas there was considerable movement between people describing “some difficulty” and “a lot of difficulty”.

**Conclusions:** Disability prevalence is high and likely to increase over time. Further research into the situation of this population is crucial to ensure inclusive policies are created and sustainable development goals are met.

## Introduction

Disability is a complex and evolving concept. The commonly-used framework for conceptualising disability is the World Health Organization (WHO) International Classification of Functioning and Disease ^1^. Essentially, a person may have a health condition (e.g. diabetes) that can cause an impairment (e.g. visual impairment), which can lead to activity limitations (e.g. difficulties walking independently) and then to participation restriction (e.g. exclusion from employment). It is not inevitable that impairments will lead to participation restriction, and this will be mediated by personal factors (e.g. wealth, education, social support) and environmental factors (e.g. accessible buildings). People with disabilities, therefore, include those with long-term physical, mental, intellectual or sensory impairments, which in interaction with various barriers may hinder their full and effective participation in society on an equal basis with others ^2^. The WHO estimates that one billion people in the world have a disability – equating to one in seven people ^3^. Of these, 110–190 million experience very significant difficulties in functioning. These numbers are expected to rise further as the global population continues to grow and average age increases. Disability is an important development issue, as the numbers affected are large, and people with disabilities face high levels of exclusion from different areas of life, such as school, employment, health and rehabilitation services ^3^, and consequently are vulnerable to poverty ^4^. It is unlikely, therefore, that the Sustainable Development Goals will be achieved without efforts to address participation exclusion among people with disabilities.

Global estimates are, however, largely based on extrapolations as data on disability are still relatively sparse. There have been global calls for research on disability: ^5^, The United Nations calls specifically for disability prevalence data using the ICF model, including through integration with national censuses and population surveys ^6^.

Moreover, there is wide variation in how disability is measured, such as whether the focus is on a specific impairment, or more holistically on participation and activities, and which tools are used. Consequently, it is difficult to compare data geographically, and over time. Consensus is growing on the use of the Washington Group (WG) Short Set to collect Disability Statistics ^7^, to improve data comparability ^8^. The WG questions focus on difficulties in six functional domains related to activities (e.g. walking) and participation (e.g. performing usual activities). These questions are increasingly being used in censuses and national surveys, but have rarely been used in prospective studies, so few measures of incidence or persistence of disability exist. Existing demographic surveillance systems throughout the world offer an opportunity to help fill the large data gaps around disability, by measuring the prevalence of disability in a population living in a defined demographic area, who are followed over time. This follow-up will allow the assessment of the long-term impacts of disability, including on survival, as well as changes over time.

Exploring disability within the context of ongoing cohorts can also help to clarify other issues, such as the association between health and disability. Overweight and obesity, hypertension, and diabetes are highly prevalent in this area of rural Malawi (9% of men and 27% of women were overweight or obese; 13% of men and 14% of women had hypertension; 2% of men and women had diabetes in a recent study) ^9^, and prevalence of NCDs is increasing over time ^10^. HIV prevalence is 9% among adults in Malawi ^11^. The occurrence of disability, by definition, requires the existence of a health condition (e.g. stroke leading to physical impairment and ultimately social exclusion). People with disabilities may also be more vulnerable to poor health, as they may be poorer, have worse health behaviours, and experience difficulties in accessing health services ^12^. Furthermore, the underlying health condition (e.g. HIV) can directly lead to disability (e.g. via hearing impairment) as well as further health conditions (e.g. metabolic syndrome). The comprehensive data collected within demographic surveillance systems can help to clarify the drivers of the complex association between health and disability.

The objectives of this study were therefore to describe the prevalence, incidence, and resolution of disability among adults in rural Malawi, and to describe the relationship between disability and chronic conditions in this cohort. Four markers of different health states were included to assess the association between disability and health: overweight and obesity, hypertension, diabetes, and HIV.

## Methods

### Setting and data collection

This study was based within the rural Karonga Health and Demographic Surveillance Site (HDSS), established in 2002 by the Malawi Epidemiology and Intervention Research Unit (MEIRU, formerly Karonga Prevention Study) in Northern Malawi. Annual censuses are taken of the population of around 42,000 individuals, collecting data on demographic, social and health indicators. There is also continuous reporting on migration, births, and deaths by informants within the community. The population is largely a subsistence-farming and fishing community and has a similar age and sex distribution to the national rural population ^13^. The WG short set questions were added onto the census in 2014 for individuals aged 18 and over. During a section of questions related to health and fertility, participants are asked the following six questions, translated into the local language of Chitumbuka:

- Do you have difficulty seeing, even if wearing glasses?
- Do you have difficulty hearing, even if using a hearing aid?
- Do you have difficulty walking or climbing steps?
- Do you have difficulty remembering or concentrating?
- Do you have difficulty (with self-care such as) washing all over or dressing?
- Using your usual (customary) language, do you have difficulty communicating, for example understanding or being understood?

For readability, they will be referred to hereafter as difficulty seeing, hearing, walking, remembering, with self-care, and communicating. For each question the participant could choose one of four possible responses: no difficulty; yes, some difficulty; yes, a lot of difficulty; and can’t do at all.

Although some HDSS census data can be collected when the participant is absent via a household proxy, the WG questions are only asked when participants are present (although they can be asked through a proxy, if the participant is unable to respond themselves, or by preference). Therefore, only those who were at home on the day of the visit provided disability data. This analysis is of the disability data from two consecutive census rounds; the first was done from 2014 to 2015 (Round 1), the second from 2015 to 2016 (Round 2). Other data relevant to this analysis collected in the Round 1 survey included age, sex, education, occupation, marital status, and proxies of socio-economic status including access to a mobile phone, and household possession score (a composite score based on value of items owned by the household).

Data on hypertension and diabetes was available from a survey of non-communicable diseases (NCDs) in adults that was performed in 2013–2015, the methods of which are described elsewhere ^14^. Blood pressure was measured three times, after 30 minutes’ rest, with 5-minute rests between measures, and the mean of the second and third readings was used in the analysis. Fasting blood glucose tests were done in the early morning after a fast of at least eight hours. All data used from this survey was taken within 2.5 years prior to the Round 1 census date.

Data on body mass index (BMI) was available from the census survey when disability data was collected. Where this was missing, BMI data was taken from the NCD survey or other studies in the same population, and the data obtained closest to the date of the Round 1 census was used. All these studies used the same procedures to measure height and weight: both are measured twice, and BMI is calculated from the mean of these measures. Of participants included in this study, 3597 (37.2%) of BMI variables came from the census survey, 5987 (62.0%) from the NCD survey, and 77 (0.8%) from other surveys. All BMI measures were taken within 3 years before or after the Round 1 census date.

Data on HIV status was collected from multiple sources: a population HIV serosurvey completed in 2011; the NCD survey; and from consenting attendees at government antiretroviral therapy (ART) clinics within the HDSS.

### Variables

We used two definitions of disability in this analysis: primarily we defined disability as participants reporting “a lot of difficulty” or “can’t do at all” in at least one of the domains asked about, as recommended by the WG ^15^; and additionally as participants reporting at least “some difficulty” in any domain. Educational attainment was grouped into; no formal education, primary education (including partially and fully completed), secondary education (including partially and fully completed), and tertiary education. Occupation was grouped into; not working, manual work (including unskilled and skilled work), farmer or fisher-man or -woman, and non-manual work (including unskilled and skilled work, professions, and businesses).

BMI was categorised as underweight (<18.5kg/m²), healthy weight (18.5–24.9kg/m²), overweight (25–24.9kg/m²), and obese (≥30kg/m²); hypertension as one or more of systolic blood pressure ≥140mmHg, diastolic blood pressure ≥90mmHg, or reported use of antihypertensive medication; and diabetes as a fasting blood glucose ≥7.0mmol/L or self-reported diagnosis of diabetes. HIV status was categorised as positive if the participant self-reported having ever had a positive HIV test, or negative if the participant had had a negative HIV test within 4 years prior to the Round 1 census date. Any negative HIV test of more than 4 years prior was counted as missing data, due to the possibility of a new HIV infection in the interim.

### Statistical analysis

We calculated the prevalence of self-reported disability by socio-demographic background stratified by sex and standardised this to the age population of the underlying census population.

We used multivariate logistic regression analysis to test for an association between BMI, hypertension, diabetes, and HIV with disability, and with each individual disability domain, controlling for age and stratified by sex. We sequentially added measures of socio-economic status including level of education, mobile phone use, and possession score to each regression model to check for confounding. No confounding was demonstrated, so we excluded them from the final models. Overweight and obesity are known risk factors for hypertension and diabetes, and we considered that BMI might also be an independent risk factor for disability. Therefore, in Model 2, we control for hypertension and diabetes to examine the relationship between BMI and disability independent of its role as a risk factor for these diseases. In Models 3 and 4, we control for BMI when examining the relationships of hypertension (Model 3) and diabetes (Model 4) with disability, as it is a potential confounder. Models 3 and 4 were run as separate models as we did not consider diabetes an *a priori* confounder in the relationship between hypertension and disability and thus excluded diabetes from Model 3, nor hypertension an *a priori* confounder in the relationship between diabetes and disability and thus excluded hypertension from Model 4.

As a sensitivity analysis, we ran a logistic regression model examining the relationship between BMI and disability excluding those with BMI measured after the census date. Secondly, we ran a model examining the relationship between BMI, hypertension, diabetes, and HIV using at least “some difficulty” as the disability outcome variable.

For those who also had disability data from Round 2, we examined the proportion whose disability status had changed between the two rounds, and calculated the incidence of disability between the two rounds (i.e. moved from no difficulty/some difficulty to a lot of difficulty/can’t do at all), and the proportion who had resolution of disability between the two rounds (i.e. moved from a lot of difficulty/can’t do at all to no difficulty/some difficulty). All analysis was done using Stata version 15.0 (StataCorp, College Station, TX).

### Ethical approval

Ethical approval for the HDSS census rounds and NCD survey was granted by the National Health Sciences Research Committee (NHSRC) (protocol numbers #419 and #1072 respectively), and by the London School of Hygiene and Tropical Medicine (LSHTM) (protocol numbers #5081 and #6303 respectively). All participants gave written informed consent to participate.

## Results

**Figure 1.**
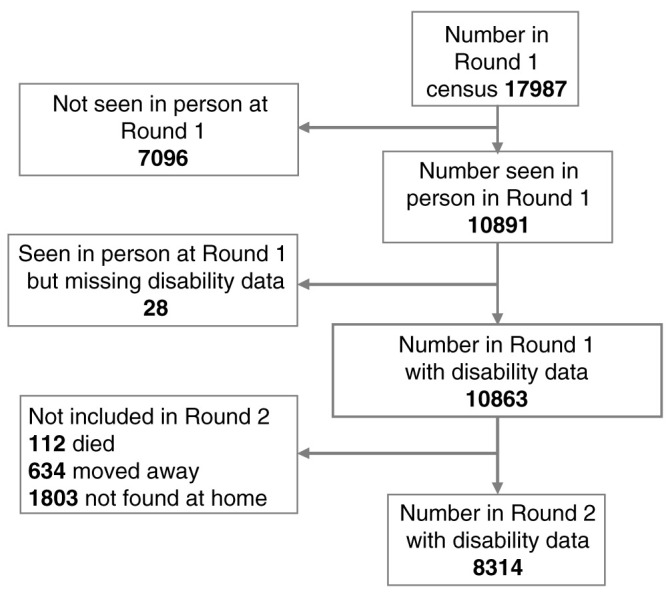
Flow chart of number of individuals participating in each round of the study.

Of 17,987 adults included in the HDSS census of 2015 (Round 1), 10,863 (60.4%) participants provided data on disability; of those who did not, 28 were seen but had missing data on disability status, and the remainder were not at home. Of those with data on disability status, 711 (6.6%) were provided by a proxy. In the Round 2 census one year later, 8,314 (76.5%) of these participants were interviewed, 112 had died, 634 had moved out of the area, and 1803 were not found at home, see Figure 1. Men were more likely to have been missed in Round 1 (58.2% of men versus 24.0% of women), as were younger participants (43.1% of the 18–39 age group versus 16.9% of the 80+ age group), shown in Table 1. There was considerable missing data on hypertension, diabetes, and HIV status. More data was missing on these health states in men than women, and among those who were not working than any other occupation group. More older people were missing data on HIV status, whereas more younger people were missing data on hypertension and diabetes. Most participants were aged under 45 and there were twice as many women as men. The most common employment for both men and women was farming or fishing (77.7% women and 68.2% men). Overweight and obesity was more common in women than men, with 28.5% of women overweight or obese compared to 10.2% of men. 15.6% of participants had hypertension, 1.9% had diabetes, and 11.9% were HIV-positive.

**Table 1.** Baseline characteristics of participants with disability data at Round 1.

	Female		Male	
	Census (n=9786)	Study (n=7437)	Census (n=8201)	Study (n=3426)
	n (%) ^1^	n (%)	n (%)	n (%)
**Age group**				
18–34	5146 (52.6)	3817 (51.3)	4478 (54.6)	1545 (45.1)
35–44	1876 (19.2)	1478 (19.9)	1599 (19.5)	711 (20.8)
45–54	1105 (11.3)	814 (10.9)	935 (11.4)	443 (12.9)
55–64	781 (8.0)	606 (8.1)	552 (6.7)	306 (8.9)
65–59	280 (2.9)	213 (2.9)	192 (2.3)	105 (3.1)
70–74	197 (2.0)	173 (2.3)	142 (1.7)	101 (2.9)
75–79	201 (2.1)	165 (2.2)	154 (1.9)	96 (2.8)
80+	200 (2.0)	171 (2.3)	149 (1.8)	119 (3.5)
Missing	0	0	0	0
**Education**				
None	378 (3.9)	296 (4.0)	104 (1.3)	69 (2.0)
Primary (part or completed)	6328 (65.1)	4980 (67.4)	3967 (48.6)	1833 (53.7)
Secondary (part or completed)	2529 (26.0)	1759 (23.8)	3449 (42.2)	1265 (37.1)
Tertiary	480 (4.9)	351 (4.8)	646 (7.9)	247 (7.2)
Missing	71	51	35	12
**Occupation**				
Not working	1152 (11.9)	621 (8.4)	1503 (18.4)	391 (11.5)
Manual work	130 (1.3)	89 (1.2)	1084 (13.3)	387 (11.3)
Farmer/fisherman	7206 (74.4)	5721 (77.7)	4643 (56.8)	2327 (68.2)
Non-manual work ^2^	1192 (12.3)	930 (12.6)	939 (11.5)	309 (9.1)
Missing	106	76	32	112
**Union status**				
Not in a union ^3^	3383 (34.6)	2343 (31.5)	2637 (32.2)	870 (25.4)
In a union	6395 (65.4)	5090 (68.5)	5552 (67.8)	2553 (74.6)
Missing	8	4	12	3
**Household possession score**				
1 (poorest)	1713 (19.7)	1399 (21.2)	1197 (16.4)	539 (17.6)
2	1469 (16.9)	1159 (17.6)	1216 (16.6)	526 (17.2)
3	1912 (22.0)	1428 (21.6)	1698 (23.3)	710 (23.2)
4	2106 (24.2)	1540 (23.3)	1903 (26.1)	812 (26.5)
5 (wealthiest)	1490 (17.1)	1075 (16.3)	1291 (17.7)	473 (15.5)
Missing	1096	836	896	366
**BMI (kg/m ^2^)** ^4^				
<18.5 (underweight)	620 (7.1)	479 (7.1)	640 (9.6)	295 (10.0)
18.5–24.9 (healthy weight)	5629 (64.9)	4314 (64.3)	5400 (80.9)	2356 (79.8)
25–29.9 (overweight)	1768 (20.4)	1398 (20.8)	550 (8.2)	266 (9.0)
30+ (obese)	658 (7.6)	518 (7.7)	83 (1.2)	35 (1.2)
Missing	1111	728	1528	474
**Hypertension** ^5^				
No hypertension	6288 (85.8)	4874 (85.3)	4641 (86.3)	2026 (82.4)
Hypertension	1041 (14.2)	837 (14.7)	737 (13.7)	434 (17.6)
Missing	2457	1726	2823	966
**Diabetes** ^6^				
No diabetes	6401 (98.2)	4990 (98.3)	4536 (98.2)	2057 (97.5)
Diabetes	117 (1.8)	87 (1.7)	81 (1.8)	53 (2.5)
Missing	3268	2360	3584	1316
**HIV status** ^7^				
Negative	6678 (88.1)	5134 (87.7)	4971 (90.4)	2164 (89.0)
Positive	902 (11.9)	719 (12.3)	527 (9.6)	267 (11.0)
Missing	2206	1584	2703	995

1. Column percentages do not include those with missing data 2. Including those working in trade and professionals 3. Including never married, divorced, and widowed 4. Calculated from the most recent height and weight measurements (all taken within 2.5 years before or after the interview date). 5. Defined as hypertension if self-report of taking anti-hypertensive medication or recorded BP=140/90 (all measured within the past 2.5 years). 6. Defined as diabetes if self-reported diagnosis or fasting blood sugar =7.0 (all measured within the past 2.5 years). 7. Defined as HIV positive if self-reported diagnosis or positive test result ever, and HIV negative if negative test result in past 4 years.

Overall crude prevalence of disability (at least “a lot of difficulty”) was 9.8% (95% CI 9.2-10.5%) in women and 9.0% (95% CI 8.1-10.0%) in men, and adjusted to the underlying population 9.5% (95% CI 8.9%-10.1%) and 8.0% (7.2%-8.9%) respectively, see Table 2. Prevalence of reporting at least “some difficulty” was 42.2% (95% CI 41.1-43.4%) in women, and 38.5% (95% CI 36.9-40.1%) in men, and adjusted to the underlying population 41.7% (95% CI 40.7-42.7%) and 35.5% (95% CI 34.1-37.0%) respectively. Prevalence of disability was higher among those with no education compared to those with education; those not working compared to those in any working category; those not in a union compared with those in a union; and similar among different household possession score groups. These relationships remain when controlling for age and sex (not shown in Tables).

**Table 2.** Prevalence of disability in any domain by socio-demographic background, stratified by sex.

	Women					Men				
		Reporting at least "some difficulty"		Reporting at least "a lot of difficulty"			Reporting at least "some difficulty"		Reporting at least "a lot of difficulty"	
	Total	n	% (95% CI)	n	% (95% CI)	Total	n	% (95% CI)	n	% (95% CI)
**Overall**										
Crude prevalence	7437	3142	42.2 (41.1-43.4)	731	9.8 (9.2-10.5)	3426	1318	38.5 (36.9-40.1)	308	9.0 (8.1-10.0)
Standardised to population structure			41.7 (40.7-42.7)		9.5 (8.9-10.1)			35.5 (34.1-37.0)		8.0 (7.2-8.9)
**Age group**										
18–34	3817	907	23.8 (22.4-25.1)	137	3.6 (3.0-4.2)	1545	321	20.8 (18.8-22.9)	48	3.1 (2.3-4.1)
35–44	1478	605	40.9 (38.5-43.5)	90	6.1 (5.0-7.4)	711	228	32.1 (28.7-35.6)	34	4.8 (3.4-6.6)
45–54	814	525	64.5 (61.1-67.7)	100	12.3 (10.2- 14.7)	443	237	53.5 (48.8-58.1)	39	8.8 (6.5-11.8)
55–64	606	454	74.9 (71.3-78.2)	97	16.0 (13.3-19.1)	306	184	60.1 (54.5-65.5)	47	15.4 (11.7-19.8)
65–69	213	176	82.6 (76.9-87.1)	49	23.0 (17.8-29.1)	105	74	70.5 (61.1-78.4)	18	17.1 (11.1-25.6)
70–74	173	163	94.2 (89.6-96.9)	68	39.3 (32.3- 46.8)	101	80	79.2 (70.2-86.0)	34	33.7 (25.1-43.4)
75–79	165	147	89.1 (83.3-93.0)	81	49.1 (41.5- 56.7)	96	83	86.5 (78.1-92.0)	34	35.4 (26.5-45.5)
80+	171	165	96.5 (92.4-98.4)	109	63.7 (56.3- 70.6)	119	111	93.3 (87.1-96.6)	54	45.4 (36.7-54.4)
**Education**										
None	296	221	74.7 (69.4-79.3)	99	33.4 (28.3-39.0)	69	50	72.5 (60.8-81.7)	19	27.5 (18.3-39.2)
Primary (part or completed)	4980	2303	46.2 (44.9-47.6)	541	10.9 (10.0-11.8)	1833	800	43.6 (41.4-45.9)	199	10.9 (9.5-12.4)
Secondary (part or completed)	1759	501	28.5 (26.4-30.6)	70	4.0 (3.2-5.0)	1265	386	30.5 (28.0-33.1)	75	5.9 (4.8-7.4)
Tertiary	351	88	25.1 (20.8-29.9)	12	3.4 (2.0-5.9)	247	77	31.2 (25.7-37.2)	14	5.7 (3.4-9.3)
**Occupation**										
Not working	621	306	49.3 (45.4-53.2)	165	26.6 (23.2-30.2)	391	138	35.3 (30.7-40.2)	78	19.9 (16.3-24.2)
Manual work	89	33	37.1 (27.7-47.5)	5	5.6 (2.4-12.8)	387	152	39.3 (34.5-44.2)	20	5.2 (3.4-7.9)
Farmer/ fisherman	5721	2430	42.5 (41.2-43.8)	491	8.6 (7.9-9.3)	2327	928	39.9 (37.9-41.9)	192	8.3 (7.2-9.4)
Non-manual work	930	335	36.0 (33.0-39.2)	53	5.7 (4.4-7.4)	309	96	31.1 (26.2-36.4)	16	5.2 (3.2-8.3)
**Union**										
Not in a union	2343	1280	54.6 (52.6-56.6)	410	17.5 (16.0-19.1)	870	257	29.5 (26.6-32.7)	69	7.9 (6.3-9.9)
In a union	5090	1862	36.6 (35.3-37.9)	321	6.3 (5.7-7.0)	2553	1060	41.5 (39.6-43.4)	239	9.4 (8.3-10.6)
**Household** **possession score**										
1 (poorest)	1,399	638	45.6 (43.0-48.2)	186	13.3 (11.6-15.2)	539	214	39.7 (35.7- 43.9)	53	9.8 (7.6-12.6)
2	1,159	490	42.3 (39.5-45.1)	109	9.4 (7.9-11.2)	526	196	37.3 (33.2- 41.5)	32	6.1 (4.3-8.5)
3	1,428	596	41.7 (39.2-44.3)	154	10.8 (9.3-12.5)	710	254	35.8 (32.3- 39.4)	61	8.6 (6.7-10.9)
4	1,540	654	42.5 (40.0-45.0)	129	8.4 (7.1-9.9)	812	328	40.4 (37.1- 43.8)	88	10.8 (8.9-13.2)
5 (wealthiest)	1,075	477	44.4 (41.4-47.4)	100	9.3 (7.7-11.2)	473	212	44.8 (40.4- 49.3)	51	10.8 (8.3-13.9)

**Figure 2.**
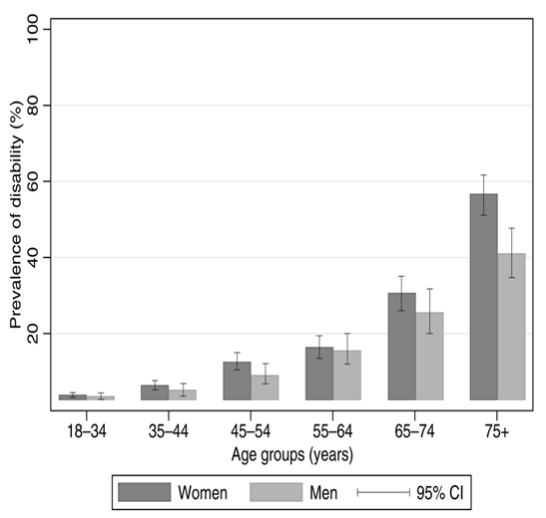
Age and sex specific prevalence of self-reported disability, defined as reporting at least “a lot of difficulty” in any domain.

The most common disabilities reported were difficulty walking at 4.5% (95% CI 4.1-4.9%) and difficulty seeing at 4.2% (95% CI 3.9-4.6%) ( *Extended Data*: Table 1). Prevalence of disability in any domain increased with age in both men and women, with 3.5% (95% CI 3.0-4.0%) of adults under age 35 reporting disability, compared to 56.2% (95% CI 50.4-61.8%) of those aged 80+, see Table 2. 24.0% (95% CI 21.5-26.7%) of adults not working reported disability versus 8.0% (95% CI 7.4-8.5%) of working adults. Figure 2 demonstrates a higher prevalence of disability in women than men in every age group, but with overlapping confidence intervals in all but the oldest age group.

We found that diabetes was associated with disability adjusted for age, as was obesity in women, whereas hypertension and HIV were not ( Table 3). The association between overweight and obesity and disability in women remained after adjusting for hypertension and diabetes. The same pattern was seen in men, but the numbers were smaller as obesity was uncommon among men and the association was not significant. In sensitivity analysis, we found that these relationships were similar when we excluded participants whose BMI was measured later than the interview date ( *Extended Data:* Table 2). Diabetes was associated with disability among men, but not women, with an OR of 2.47 (95% CI 1.32-4.64) adjusted for age, which remained after adjusting for BMI. The association between obesity and disability was driven by a strong association with difficulty walking (OR 2.78; 95% CI 1.94-3.98), and diabetes was associated with difficulty seeing (OR 2.28; 95% CI 1.39-3.73). Hypertension was also associated with difficulty walking, but overall was not associated with disability ( *Extended Data:* Table 3).

**Table 3.** Logistic regression analysis of the association between obesity, hypertension, diabetes, and HIV with self-reported disability.

			Bivariate analysis – Models 1A & 1B *		Multivariate analysis – Models 2A & 2B **		Multivariate analysis – Models 3A & 3B ***		Multivariate analysis – Models 4A & 4B ****	
		Number reporting difficulty	OR (95% CI)	p-value	OR (95% CI)	p-value	OR (95% CI)	p-value	OR (95% CI)	p-value
**BMI (kg/m ^2^)**										
Women	<18.5	51/479	0.84 (0.65-1.09)	0.005	0.66 (0.43-1.01)	0.005				
	18.5-24.9	356/4314	1		1					
	25-29.9	152/1398	1.24 (1.03-1.50)		1.28 (1.00-1.63)					
	30+	78/518	1.40 (1.07-1.83)		1.39 (1.01-1.92)					
Men	<18.5	41/295	1.16 (0.78-1.70)	0.10	1.20 (0.75-1.93)	0.16				
	18.5-24.9	184/2356	1		1					
	25-29.9	37/266	1.36 (0.91-2.03)		1.21 (0.75-1.96)					
	30+	9/35	2.62 (1.14-6.02)		2.93 (1.15-7.46)					
**Hypertension**										
Women	No hypertension	366/4874	1	0.80			1	0.40		
	Hypertension	215/837		1.03 (0.82-1.30)			0.90 (0.71-1.15)			
Men	No hypertension	144/2026	1	0.13			1	0.28		
	Hypertension	90/434	1.30 (0.93-1.81)				1.21 (0.86-1.71)			
**Diabetes**										
Women	No diabetes	478/4990	1	0.37					1	0.09
	Diabetes	20/87	1.29 (0.74-2.24)						1.11 (0.63-1.97)	
Men	No diabetes	190/2057	1	0.007					1	0.003
	Diabetes	18/53	2.47 (1.32-4.64)						2.37 (1.25-4.52)	
**HIV**										
Women	HIV negative	454/5134	1	0.32						
	HIV positive	56/719	0.86 (0.64-1.16)							
Men	HIV negative	188/2164	1	0.83						
	HIV positive	26/267	1.05 (0.67-1.65)							

*Model 1: Bivariate analysis adjusted for age only (as linear variable). Model 1A included women only, Model 1B included men only. **Model 2: Models adjusted for age (as a linear variable), hypertension (hypertension/ no hypertension/ unknown), and diabetes (diabetes/ no diabetes/ unknown diabetes). Model 2A included women only, Model 2B included men only. ***Model 3: Models adjusted for age (as a linear variable) and BMI (<18.5 kg/m ^2^/ 18–24.9 kg/m ^2^/ 25–29.9 kg/m ^2^/ 30+kg/m ^2^). Model 3A included women only, Model 3B included men only. ****Model 4: Models adjusted for age (as a linear variable) and BMI (<18.5 kg/m ^2^/ 18–24.9 kg/m ^2^/ 25–29.9 kg/m ^2^/ 30+kg/m ^2^). Model 4A included women only, Model 4B included men only.

When using reports of at least “some difficulty” as the disability outcome in sensitivity analysis, the relationship between BMI and disability in women remained: women with higher BMI had more than twice the odds of reporting disability than those with healthy BMI *(Extended Data:* Table 4 *)*; but there was no association between diabetes and disability.

**Figure 3.**
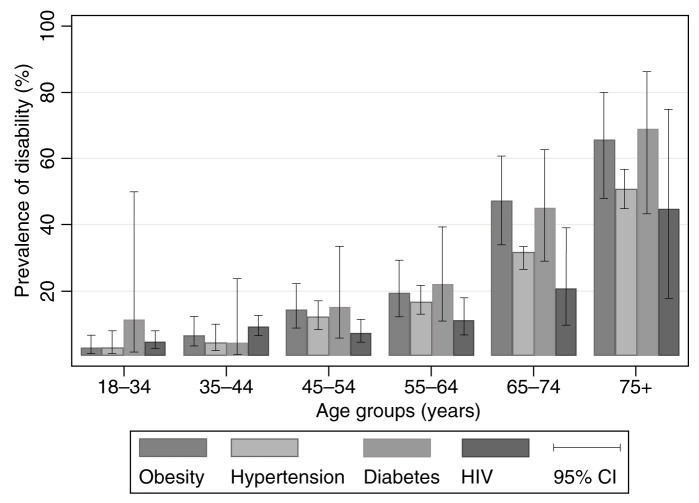
Age-specific prevalence of self-reported disability with obesity, hypertension, diabetes, and HIV-infection.

Figure 3 shows that age-specific prevalence of disability appeared to be higher with obesity and diabetes than with hypertension, and lower with HIV-infection than any of the other conditions examined. However, the confidence intervals were overlapping.

Table 4 shows self-reported disability status in each domain over the two rounds. Incident disability was between 0.3% (95% CI 0.2-0.4%) for difficulty communicating and 2.3% (95% CI 2.0-2.6%) for difficulty walking. Between 61.9% (95% CI 40.2-79.7%) to 90.6% (95% CI 85.4-94.1%) of disability resolved between the two rounds. Of those who reported “no difficulty” or “can’t do at all” at Round 1, the majority stayed within the same category at Round 2. However, most people who reported “some difficulty” or “a lot of difficulty” changed category, usually with an improvement in functional status (i.e. less disability). Of participants reporting “some difficulty” in any domain in Round 1, 44.5-80.3% reported “no difficulty” the following year in that domain; of those reporting “a lot of difficulty” in Round 1, 26.7-75.0% reported “no difficulty” the following year Those aged under 60 were more likely to report an improved functional status at Round 2 compared to those aged over 60 ( *Extended Data:* Table 5).

**Table 4.** Panel data of reported disability over time, including incidence and resolution of disability.

	Round 1	No difficulty	Some difficulty	A lot of difficulty	Can’t do at all	Incident disability	Resolution of disability
**Difficulty seeing**		n % (95% CI)	n % (95% CI)	n % (95% CI)	n % (95% CI)	n % (95% CI)	n % (95% CI)
	**No** **difficulty**	5719/6282 **91.0%** (90.3-91.7%)	519/6282 **8.3%** (7.6-9.0%)	44/6282 **0.7%** (0.5-0.9%)	0/6282 **0%**	137/7921 **1.7%** (1.5-2.0%)	
	**Some** **difficulty**	730/1639 **44.5 %** (42.2-47.0%)	816/1639 **49.9%** (47.4-52.2%)	91/1639 **5.6%** (4.5-6.8%)	2/1639 **0.1%** (0.03-0.5%)		
	**A lot of** **difficulty**	98/367 **26.7%** (22.4-31.5%)	205/367 **55.9%** (50.7-60.9%)	52/367 **14.2%** (11.0-18.1%)	12/367 **3.3%** (1.9-5.7%)		305/388 **78.6%** (74.2-82.4%)
	**Can't do at** **all**	0/21 **0%**	2/21 **9.5%** (2.4-31.1%)	2/21 **9.5%** (2.4-31.1%)	17/21 **81.0%** (58.8-92.7%)		
**Difficulty hearing**	**No** **difficulty**	7644/7848 **97.4%** (97.0-97.7%)	189/7848 **2.4%** (2.1-2.8%)	14/7848 **0.2%** (0.1-0.3%)	1/7848 **0.01%** (0-0.09%)	37/8233 **0.4%** (0.3-0.6%)	
	**Some** **difficulty**	229/385 **59.5%** (54.5-64.3%)	134/385 **34.8%** (30.2-39.7%)	20/385 **5.2%** (3.4-7.9%)	2/385 **0.52%** (0.1-2.1%)		
	**A lot of** **difficulty**	19/66 **28.8%** (19.2-40.8%)	29/66 **43.9%** (32.5-56.0%)	16/66 **24.2%** (15.4-36.0%)	2/66 **3.0%** (0.8-11.3%)		48/75 **64.0%** (52.6-74.0%)
	**Can't do at** **all**	0/9 **0%**	0/9 **0%**	3/9 **33.3%** (11.1-66.7%)	6/9 **66.7%** (33.3-88.9%)		
**Difficulty walking**	**No** **difficulty**	6276/6771 **92.7%** (92.0-93.3%)	434/6771 **6.4%** (5.9-7.0%)	57/6771 **0.8%** (0.7-1.1%)	4/6771 **0.06%** (0.02-0.2%)	181/7912 **2.3%** (2.0-2.6%)	
	**Some** **difficulty**	618/1141 **54.2%** (51.3-57.0%)	403/1141 **35.3%** (32.6-38.1%)	118/1141 **10.3%** (8.7-12.3%)	2/1141 **0.2%** (0.04-0.7%)		
	**A lot of** **difficulty**	126/373 **33.8%** (29.2-38.7%)	151/373 **40.5%** (35.6-45.6%)	92/373 **24.7%** (20.6-29.3%)	4/373 **1.1%** (0.4-2.8%)		279/375 **70.6%** (65.9-74.9%)
	**Can't do at** **all**	2/22 **9.1%** (2.3-30.0%)	0/22 **0%**	6/22 **27.3%** (12.8-48.9%)	14/22 **63.6%** (42.3-80.7%)		
**Difficulty** **remembering**	**No** **difficulty**	6011/6631 **90.7%** (89.9-91.3%)	564/6631 **8.5%** (7.9-9.2%)	55/6631 **0.8%** (0.6-1.1%)	1/6631 **0.02%** (0-0.1%)	109/8109 **1.3%** (1.1-1.6%)	
	**Some** **difficulty**	1083/1478 **73.3%** (71.0-75.5%)	342/1478 **23.1%** (21.1-25.4%)	52/1478 **3.5%** (2.7-4.6%)	1/1478 **0.07%** (0.01-0.5%)		
	**A lot of** **difficulty**	108/180 **60.0%** (52.7-66.9%)	56/180 **31.1%** (24.8-38.2%)	13/180 **7.2%** (4.2-12.0%)	3/180 **1.7%** (0.5-5.0%)		164/181 **90.6%** (85.4-94.1%)
	**Can't do at** **all**	0/1 **0%**	0/1 **0%**	1/1 **100%**	0/1 **0%**		
**Difficulty** **communication**	**No** **difficulty**	8086/8184 **98.8%** (98.5-99.0%)	81/8184 **1.0%** (0.8-1.2%)	12/8184 **0.2%** (0.08-0.3%)	5/8184 **0.06%** (0.03-0.2%)	22/8264 **0.3%** (0.2-0.4%)	
	**Some** **difficulty**	62/80 **77.5%** (67.1-85.3%)	13/80 **16.3%** (9.7-26.0%)	5/80 **6.3%** (2.6-14.2%)	0/80 **0%**		
	**A lot of** **difficulty**	9/16 **56.3%** (32.4-77.5%)	3/16 **18.8%** (6.2-44.8%)	2/16 **12.5%** (3.1-38.6%)	2/16 **12.5%** (3.1-38.6%)		13/21 **61.9%** (40.2-79.7%)
	**Can't do at** **all**	1/5 **20.0%** (2.7-69.1%)	0/5 **0%**	1/5 **20.0%** (2.7-69.1%)	3/5 **60.0%** (20.0-90.0%)		
**Difficulty with self-** **care**	**No** **difficulty**	7434/7759 **95.8%** (95.3-96.2%)	273/7759 **3.5%** (3.1-4.0%)	46/7759 **0.6%** (0.4-0.8%)	6/7759 **0.08%** (0.03-0.2%)	68/8134 **0.8%** (0.7-1.1%)	
	**Some** **difficulty**	301/375 **80.3%** (75.9-84.0%)	58/375 **15.5%** (12.2-19.5%)	13/375 **3.5%** (2.0-5.9%)	3/375 **0.8%** (0.3-2.5%)		
	**A lot of** **difficulty**	111/148 **75.0%** (67.4-81.3%)	24/148 **16.2%** (11.1-23.1%)	12/148 **8.1%** (4.7-13.7%)	1/148 **0.7%** (0.1-4.6%)		139/159 **87.4%** (81.3-91.7%)
	**Can't do at** **all**	2/11 **18.2%** (4.6-50.7%)	2/11 **18.2%** (4.6-50.7%)	5/11 **45.5%** (20.3-73.2%)	2/11 **18.2%** (4.6-50.7%)		

Legend: Self-reported disability status of participants at Round 2, according to their status at Round 1, for all disability domains

## Discussion

Around one in ten study participants reported disability, most commonly difficulty walking or seeing. Prevalence was higher in women than men and increased rapidly with age, with one in four adults over 50 reporting disability. While obesity and diabetes were associated with self-reported disability (obesity with women only), hypertension and HIV were not. Reporting severe levels of disability (“can’t do at all”) in a functional domain was relatively consistent between the two rounds, whereas most of those who reported “some difficulty” or “a lot of difficulty” at Round 1 reported a changed disability category at Round 2, one year later.

Direct comparison of prevalence with other studies is challenging, even when the WG questions have been used, as population age distribution has a strong impact on prevalence and varies between sites, and age-specific prevalence is not usually presented. In 2010–2011, the Washington Group short set questions were asked to adults aged 15 and over in the Malawi Integrated Household Survey, where a much lower prevalence of disability was found in every age category: overall 1.4% of people had at least “a lot of difficulty” in at least one domain. The difference may be explained by a different proportion of responses given by a proxy (as proxy respondents are likely to underestimate the prevalence of functional difficulties), or differences in the way the survey questions were posed ^5^.

The discourse on disability in low and middle income countries (LMICs) links disability closely with poverty ^16– 18^. Therefore, in Malawi, a poor and food-insecure country ^19^, disability might be expected to be associated with under-nutrition and low BMI, particularly as research has previously demonstrated obesity to be more common in people from less poor households in this population ^9^. While we found that disability was associated with having no education and not working (both associated with financial insecurity and poverty), we found no association between disability and household possession score. Our findings did demonstrate a stepwise increase in odds of disability with increasing BMI however, particularly among women, independent of hypertension and diabetes, and was present for both disability as defined by at least “a lot of difficulty” and at least “some difficulty”. This association was mainly driven by difficulty walking, which may suggest that obesity is a consequence of lack of exercise secondary to disability, or that obesity has led to disabling complications such as osteoarthritis ^20^. Obesity is well-recognised to be associated with disability in high income countries (HIC) ^21– 23^, but this association has only rarely been seen in LMICs ^24, 25^. Similarly, while disability is strongly associated with diabetes in HIC ^26– 28^, evidence in LMICs has been less consistent ^29– 31^. Difficulty seeing in diabetes is likely to be secondary to diabetic eye disease, and perhaps in this rural Malawian setting, where diabetes is frequently undiagnosed and sub-optimally controlled ^9^, eye disease develops early in the disease course ^32^. Both obesity and diabetes are highly prevalent ^9^, and should be recognised as potentially important drivers for disability among this population. This may be the first study investigating the relationship between disability and disease states through biomarkers other than HIV infection in LMICs ^33^. Systematic reviews of the association of disability with BMI, hypertension and diabetes in LMICs would be a useful contribution to the literature to hep elucidate these associations further.

Our finding that hypertension was not associated with disability was in keeping with a meta-analysis analysing the contribution of chronic diseases to disability in older people in LMICs ^31^. The literature on HIV infection and disability in sub-Saharan Africa is mixed: HIV has been shown to be associated with frailty ^34, 35^, a syndrome closely linked to disability ^36^, and a systematic review found that in 27 of 37 studies, people living with HIV had lower levels of functioning than those without HIV ^34^. However, the data did not allow disaggregation by use of anti-retroviral therapy, and the association between HIV and disability may have changed over time as antiretroviral availability has improved ^37^.

Our estimates of the percentage of people reporting disability at Round 1 experiencing a resolution of disability by Round 2 were high, between 61.9% and 90.6%. This is mainly explained by considerable movement between those reporting “a lot of difficulty” and “some difficulty” with many participants reporting an improved functional status, and less disability, the following year. This is likely to represent both an element of true fluctuation of disability and changing descriptions of a constant level of disability over time. Our study found that most people who reported “can’t do at all” for any domain at Round 1 consistently reported disability at Round 2. This movement of people in and out of disability status was also seen from similar panel data using the Washington Group short set in Ethiopia and Uganda ^5^. While some people did move from “can’t do at all” to “no difficulty” in the domains of walking, communicating, and self-care, the numbers were very small and this may have represented acute illness that resolved.

Our study has some important strengths. Due to the large sample size and collection of data on other health states, we can obtain precise estimates of disability prevalence and examine associations between health and disability. Collecting data on disability and chronic conditions at different contacts reduces the likelihood of spurious self-report or observer bias. As further rounds of census data are collected, we will be able to analyse trends over time and further understanding about the trajectories of disability prevalence in this context.

There are some inherent limitations to self-report of disability, particularly in certain domains. People with difficulty hearing or communicating may have challenges interacting with the interviewer, and people with difficulty remembering may lack insight into their difficulties. However, self-report does allow a reflection of an individual’s lived experiences of disability more than clinical assessment of impairment or function. Comparisons of self-reported disability between age-groups, sexes, and externally to other populations may be less valid than when using clinical assessment of impairments as there may be cultural differences between willingness to report disability or different levels of stoicism (or expected function) ^38, 39^. Furthermore, the WG questions do not capture a complete picture of disability, as they do not include pain or low mood and focus more on functional limitations than participation. However, the brevity of the WG questions does allow the questions to be easily added to existing surveys. The WG recommends defining disability as at least “a lot of difficulty”. However, if participants’ descriptions of constant disabilities do vary over time between “some difficulty” (categorised as no disability) and “a lot of difficulty” (categorised as having disability), using this cut-off may lead to measurement error and imprecision in estimates of associations and trends.

Our missing data for those absent from home at the time of survey, particularly younger men, may have led to an over-estimate of disability prevalence, as this group is likely to have a lower disability prevalence than those at home. Conversely, some people with disability may have been excluded from the survey, for example if they were hidden, in residential care, or away seeking healthcare. We are also missing data on HIV status, hypertension, and diabetes for substantial numbers of participants, and for HIV, we were more likely to capture positive than negative diagnoses as data was partly gathered from participants attending HIV clinics ^14^. This may have introduced some bias into our analysis of the association between chronic disease and disability. There may also be some survival bias: among those who were diagnosed with chronic disease such as HIV in the three years prior to the survey, those with the most advanced disease may have died in the interim. Our participants may therefore represent those with less severe disease, which may be less likely to be associated with disability. This would lead to a false attenuation of the relationship between chronic disease and disability, and the relationships may be even stronger than we estimated.

## Conclusion

Self-reported disability prevalence in this area of rural Malawi is around 10% in adults, and even in this very poor rural setting there are significant independent associations between obesity and disability in women, and diabetes and disability in both sexes , both of which are already a considerable burden in this population. Combined with an ageing and expanding population the number of people living with disability is likely to increase significantly over the coming years. Further investigation into the needs of this potentially vulnerable population is vital in order to create inclusive public health and social policies.

## Data availability

### Underlying data

LSHTM Data Compass: Malawi Epidemiology and Intervention Research Unit Non-Communicable Disease Survey data, 2013–2017. https://doi.org/10.17037/DATA.00000961 ^40^. Data are available under the terms of the Creative Commons Attribution 3.0 International license (CC-BY 3.0).

Summary demographic datasets are publicly available through the INDEPTH iShare platform.

Longitudinal data (demographic surveillance episodes and linked rounds of disability questionnaires) cannot be sufficiently de-identified for public availability. Application may be made for access through the MEIRU director ( mia.crampin@lshtm.ac.uk) or data scientist Chifundo Kanjala ( chifundo.kanjala@lshtm.ac.uk). Those wishing to access the data will need to provide a brief proposal for what the data will be used for as a condition of access.

### Extended data

Harvard Dataverse: Self-reported disability in rural Malawi: prevalence, incidence, and relationship to BMI and chronic disease: Extended Data. https://doi.org/10.7910/DVN/IAELBG ^41^.

This project contains the following extended data:

1. Extended Data Table 1: Prevalence (%) of self-reported disability in each disability domain by age at Round 1
2. Extended Data Table 2: Logistic regression analysis of the association between BMI and self-reported disability (excluding BMI measurements taken after the date of the study interview)
3. Extended Data Table 3: Logistic regression analysis of the association between BMI, hypertension, diabetes, and HIV with self-reported disability in different domains at Round 1
4. Logistic regression analysis of the association between BMI, hypertension, diabetes, and HIV infection with self-reported disability in any domain at Round 1 (disability defined as at least “some difficulty” in any domain)
5. Extended Data Table 3: Self-reported disability status at Round 2, according to their status at Round 1, for all disability domains stratified by age group

Data are available under the terms of the Creative Commons Zero “No rights reserved” data waiver (CC0 1.0 Public domain dedication).
