# Supplementary material for: Self-reported disability in rural Malawi: prevalence, incidence, and relationship to chronic conditions
Source: Wellcome Open Res. 2021 Aug 27;4:90. Originally published 2019 Jun 7. [Version 5] doi: 10.12688/wellcomeopenres.15196.5 (PMC7722531; doi:10.12688/wellcomeopenres.15196.5)
Supplement: Peer review history

# Peer review: Reviewer response for version 3

**DOI:** 10.21956/wellcomeopenres.18019.r41023

**Published:** 2020-12-07

**Authors:** Nawi Ng

**Affiliations:** 1 School of Public Health and Community Medicine Institution of Medicine, Sahlgrenska Academy, University of Gothenburg, Gothenburg, Sweden

The authors have addressed all my comments in the previous rounds. Congratulations for the good work.

Is the work clearly and accurately presented and does it cite the current literature?

Partly

If applicable, is the statistical analysis and its interpretation appropriate?

No

Are all the source data underlying the results available to ensure full reproducibility?

No

Is the study design appropriate and is the work technically sound?

Partly

Are the conclusions drawn adequately supported by the results?

Partly

Are sufficient details of methods and analysis provided to allow replication by others?

No

Reviewer Expertise:

Ageing and disability, risk factor surveillance, epidemiology analysis, longitudinal data analysis.

I confirm that I have read this submission and believe that I have an appropriate level of expertise to confirm that it is of an acceptable scientific standard.

# Peer review: Reviewer response for version 3

**DOI:** 10.21956/wellcomeopenres.18019.r41024

**Published:** 2020-11-04

**Authors:** Sophie Mitra

**Affiliations:** 1 Fordham University, New York, NY, USA

Thank you for the revised version. The paper has significantly improved.

 One detail though still need to be addressed. It was point 5 in my earlier review: 

 For the Malawi results using the Malawi Integrated Household Survey 2010/11 and for the Uganda and Ethiopia results on changes in disability status over time, the correct reference is Mitra S: Disability, Health and Human Development. Palgrave MacMillan: New York, ***and not ***the article in AJPH.

Is the work clearly and accurately presented and does it cite the current literature?

Yes

If applicable, is the statistical analysis and its interpretation appropriate?

Yes

Are all the source data underlying the results available to ensure full reproducibility?

Yes

Is the study design appropriate and is the work technically sound?

Partly

Are the conclusions drawn adequately supported by the results?

Partly

Are sufficient details of methods and analysis provided to allow replication by others?

Partly

Reviewer Expertise:

Disability; economics; public health.

I confirm that I have read this submission and believe that I have an appropriate level of expertise to confirm that it is of an acceptable scientific standard.

# Peer review: Reviewer response for version 2

**DOI:** 10.21956/wellcomeopenres.17087.r37256

**Published:** 2019-12-12

**Authors:** Nawi Ng

**Affiliations:** 1 School of Public Health and Community Medicine Institution of Medicine, Sahlgrenska Academy, University of Gothenburg, Gothenburg, Sweden

Thank you for the invitation to review this revised manuscript. I commend the authors’ effort in revising and improving the quality of the paper. As much as I would like to support this research to be indexed, I still have significant concern about the quality of the paper. 

**The validity of self-reported disability data: **I appreciate the authors’ effort to assess the “consistency” of self-reported disability data collected in Round 1 and Round 2 of the disability survey (one year apart). By comparing response patterns in Round 1 and Round 2, the authors estimated the incidence of disability and resolution of disability. If the reporting of disability is valid and reliable, these estimates will give a picture of how disability changes over time. Table 4, in contrast, shows that the self-reported disability seems to be inconsistent over time. In my initial review, I raised the concern about 9.1% of respondents who could not walk at all in the baseline but reported no difficulty in the follow-up. The authors argued that these represent only 2 out of 22 individuals, hence is understandable. However, how could we interpret the data in Table 4 which show 78.6% of those with a lot of difficulty in seeing (the question asks, “Do you have difficulty seeing, **even if wearing glasses**?”) had their seeing difficulty resolved within one year? The same pattern was observed in other domains of disability. These findings are unexpected in rural Malawi which, according to the authors, was a “very poor rural setting” where one does not expect interventions of any kinds to happen during the study period. By the way, the term “consistency” still appears in the objective (in Abstract and main text) and the title for Table 4.

 Lack of clarity in the multivariate analyses (Table 3): There are two issues about Table 3 that I could not understand. **Firstly**, according to the authors, Model 2 is a multivariate model regressing disability on body mass index and controlling for hypertension, diabetes and age. Model 3 is a multivariate model regression disability on hypertension and diabetes and controlling for body mass index and age. Aren’t these two models the same model? Why were they referred to as different models? **Secondly**, it is unclear how the authors could obtain the results for BMI, hypertension and diabetes separately for men and women in a single model (Model 2 or Model 3). The results in Table 3 could only be obtained by stratifying the analysis by sex (if so, the model should be referred to as Model 2A for men and Model 2B for women). Even if the authors had included an interaction term between sex and BMI (not declared in the paper), they would have only one reference category, not two as reported in Table 3 (BMI 18.5-24.9 for men and women). In brief, it is difficult to understand what Table 3 represents, and this puts doubt on the main findings and the conclusion of this study.  

**Potential biases influencing the conclusions of the study:** I appreciate the effort of the authors to link data from multiple sources collected 1-3 years before the baseline survey of disability. In my earlier review, I raised the concern about missing data and selection bias due to missing not at random. It is insufficient to acknowledge these issues as a limitation of the study, especially when the potentially biased results could influence the conclusion of the study. How sure could the authors conclude that hypertension and HIV are not associated with disability, knowing that those with severe hypertension or late-stage HIV (measured a few years before the disability survey) might have died already before the disability survey started? Hence, those who participated in the survey had less severe hypertension or early stage of HIV, and hence experienced less severe disability (survival bias).

 A minor issue on the data linkage – how did the researchers handle the data, for example, the body mass index, if the individual data on BMI was missing from the census survey data but existed in more than one other studies (it could happen). Which data value was considered in the current study?

Is the work clearly and accurately presented and does it cite the current literature?

Partly

If applicable, is the statistical analysis and its interpretation appropriate?

No

Are all the source data underlying the results available to ensure full reproducibility?

No

Is the study design appropriate and is the work technically sound?

Partly

Are the conclusions drawn adequately supported by the results?

Partly

Are sufficient details of methods and analysis provided to allow replication by others?

No

Reviewer Expertise:

Ageing and disability, risk factor surveillance, epidemiology analysis, longitudinal data analysis.

I confirm that I have read this submission and believe that I have an appropriate level of expertise to state that I do not consider it to be of an acceptable scientific standard, for reasons outlined above.

# Response

**Published:** 2020-10-12

**Authors:** Josephine Prynn

**Affiliations:** University College London, UK

Dear Professor Ng,

Many thanks for your thoughtful comments, and apologies for the delay in responding to them. We have submitted an updated manuscript that addresses your concerns and suggestions. In particular we have clarified our use of panel data in Table 4 to estimate incidence and resolution of disability over time, and have updated the discussion regarding these results. We have also amended Table 3 to clarify our use of bivariate and multivariate regression, I hope that this is now clearer. We have also updated our Discussion to explore the possible effect of survival bias. 

We address each of your individual points below: 

**The validity of self-reported disability data: I appreciate the authors’ effort to assess the “consistency” of self-reported disability data collected in Round 1 and Round 2 of the disability survey (one year apart). By comparing response patterns in Round 1 and Round 2, the authors estimated the incidence of disability and resolution of disability. If the reporting of disability is valid and reliable, these estimates will give a picture of how disability changes over time. Table 4, in contrast, shows that the self-reported disability seems to be inconsistent over time. In my initial review, I raised the concern about 9.1% of respondents who could not walk at all in the baseline but reported no difficulty in the follow-up. The authors argued that these represent only 2 out of 22 individuals, hence is understandable. However, how could we interpret the data in Table 4 which show 78.6% of those with a lot of difficulty in seeing (the question asks, “Do you have difficulty seeing, even if wearing glasses?”) had their seeing difficulty resolved within one year? The same pattern was observed in other domains of disability. These findings are unexpected in rural Malawi which, according to the authors, was a “very poor rural setting” where one does not expect interventions of any kinds to happen during the study period. By the way, the term “consistency” still appears in the objective (in Abstract and main text) and the title for Table 4.**

Many thanks for this observation. We have now changed the wording of the discussion to emphasise that most of the resolution was from “a lot” to “some” difficulty.  We have also removed the word “consistency” from the abstract, main text and table in order to emphasise that the main aim of this analysis was to examine the incidence and resolution of disability over time. We acknowledge however, that some of this change will be due to differing descriptions of the same disability over time rather than change in function, and have kept that explanation in the Discussion. We have removed the statement that interventions might explain some of the resolution as pointed out this is unlikely to be a major contributory factor.

**Lack of clarity in the multivariate analyses (Table 3): There are two issues about Table 3 that I could not understand. Firstly, according to the authors, Model 2 is a multivariate model regressing disability on body mass index and controlling for hypertension, diabetes and age. Model 3 is a multivariate model regression disability on hypertension and diabetes and controlling for body mass index and age. Aren’t these two models the same model? Why were they referred to as different models? Secondly, it is unclear how the authors could obtain the results for BMI, hypertension and diabetes separately for men and women in a single model (Model 2 or Model 3). The results in Table 3 could only be obtained by stratifying the analysis by sex (if so, the model should be referred to as Model 2A for men and Model 2B for women). Even if the authors had included an interaction term between sex and BMI (not declared in the paper), they would have only one reference category, not two as reported in Table 3 (BMI 18.5-24.9 for men and women). In brief, it is difficult to understand what Table 3 represents, and this puts doubt on the main findings and the conclusion of this study.**

Apologies that the way we had labelled the Models was not clear. We hope that our analysis is clearer now: as the analysis was stratified by sex, we have clarified that the models for women and men were different by calling them Models A and B.  We have also added a separate column for Model 4 (regressing disability on hypertension controlling for BMI) to separate it from Model 3 (regressing disability on diabetes controlling for BMI).   We have updated text in the Methods section to explain that Models 3 and 4 were actually different.  

**Potential biases influencing the conclusions of the study: I appreciate the effort of the authors to link data from multiple sources collected 1-3 years before the baseline survey of disability. In my earlier review, I raised the concern about missing data and selection bias due to missing not at random. It is insufficient to acknowledge these issues as a limitation of the study, especially when the potentially biased results could influence the conclusion of the study. How sure could the authors conclude that hypertension and HIV are not associated with disability, knowing that those with severe hypertension or late-stage HIV (measured a few years before the disability survey) might have died already before the disability survey started? Hence, those who participated in the survey had less severe hypertension or early stage of HIV, and hence experienced less severe disability (survival bias).**

We recognise that this was an issue, and we have updated the Discussion to reflect this and add further detail to the effect it may have had on the results.

**A minor issue on the data linkage – how did the researchers handle the data, for example, the body mass index, if the individual data on BMI was missing from the census survey data but existed in more than one other studies (it could happen). Which data value was considered in the current study?**

The data collected closest to the date of the survey was used.  We have clarified this in the text.

Many thanks again,

Josephine Prynn

# Peer review: Reviewer response for version 2

**DOI:** 10.21956/wellcomeopenres.17087.r37255

**Published:** 2019-12-09

**Authors:** Sophie Mitra

**Affiliations:** 1 Fordham University, New York, NY, USA

The paper has very much improved. It is more focused and clearly written and makes a significant contribution to the literature. 

 There are a few things that need to be revised.

1. The introductory background is very useful. A few sentences and references on the prevalence of chronic conditions in Malawi, for instance on the recent rise in obesity would add to the useful background. 
2. The results are presented as applying to all of Malawi. This is for instance the case in the abstract and in the conclusion starts with “Self-reported disability prevalence in rural Malawi is around 10% in adults”. Yet, the data under use is not nationally representative. The authors need to carefully edit so that the results are presented as for this study, based within the rural Karonga Health and Demographic Surveillance Site.
3. I recommend that Table 1 includes descriptive statistics for all variables used in the study, including obesity.
4. The authors do not give results on the association between disability and socioeconomic status, which as they note, has been an important issue in the literature. At least, a mention of their results on this, would be useful. This is particular in light of the association found between obesity and disability for women. 
5. For the Malawi results using the Malawi Integrated Household Survey 2010/11 and for the Uganda and Ethiopia results on changes in disability status over time, the correct reference is Mitra S: Disability, Health and Human Development. Palgrave MacMillan: New York, and not the article in AJPA. 
6. Regarding the Malawi results using the Malawi Integrated Household Survey 2010/11, the authors of this paper mention the potentially different age structures of the population in the national survey compared to their survey. They should actually check on that given the results in the book above (Table 4.4).
7. The wording of the abstract needs to be carefully edited for accuracy. For instance, the association of obesity and disability is found only for women, but is presented as a general result in the abstract. 
8. The authors note that “the WG questions do not capture a complete picture of disability, as they do not include pain or low mood and focus more on functional limitations than participation. Our use of this tool therefore meant that we were not able to assess participation restriction, which is a fundamental component of the ICF model of disability. However, the brevity of the WG questions does allow the questions to be easily added to existing surveys, and we did also report on employment inclusion and marital status as proxies for participation”. The WG questions combined with other questions for instance on employment can be used to assess participation restrictions. Hence, this text is unnecessary. It was not within the scope of the paper to assess participation restriction, which is okay.
9. Sometimes in the text, LMIC need to be switched to plural.

Is the work clearly and accurately presented and does it cite the current literature?

Yes

If applicable, is the statistical analysis and its interpretation appropriate?

Yes

Are all the source data underlying the results available to ensure full reproducibility?

Yes

Is the study design appropriate and is the work technically sound?

Partly

Are the conclusions drawn adequately supported by the results?

Partly

Are sufficient details of methods and analysis provided to allow replication by others?

Partly

Reviewer Expertise:

Disability; economics; public health.

I confirm that I have read this submission and believe that I have an appropriate level of expertise to confirm that it is of an acceptable scientific standard, however I have significant reservations, as outlined above.

# Response

**Published:** 2020-10-12

**Authors:** Josephine Prynn

**Affiliations:** University College London, UK

Dear Professor Mitra,

Many thanks for your helpful and thoughtful comments. We have re-submitted an updated manuscript which addresses your suggestions and concerns. In particular we have updated the abstract and conclusion to clarify that our findings are from one DHS and not necessarily applicable to the whole country; we have added some more information on the prevalence of chronic conditions in Malawi; and have clarified our findings on the relationship between disability and socio-economic status. 

I have responded to each of your points in turn below. 

**The introductory background is very useful. A few sentences and references on the prevalence of chronic conditions in Malawi, for instance on the recent rise in obesity would add to the useful background.**

Thank you for this suggestion. This has now been added to the introduction.

**The results are presented as applying to all of Malawi. This is for instance the case in the abstract and in the conclusion starts with “Self-reported disability prevalence in rural Malawi is around 10% in adults”. Yet, the data under use is not nationally representative. The authors need to carefully edit so that the results are presented as for this study, based within the rural Karonga Health and Demographic Surveillance Site.**

We have updated the Abstract and Conclusion to clarify that this data does not represent the whole of Malawi.

**I recommend that Table 1 includes descriptive statistics for all variables used in the study, including obesity.**

Table 1 includes the variable of BMI, including the category of obesity. We chose to include all BMI categories to show the proportion of people who were underweight as well as overweight and obese.

**The authors do not give results on the association between disability and socioeconomic status, which as they note, has been an important issue in the literature. At least, a mention of their results on this, would be useful. This is particular in light of the association found between obesity and disability for women.**

Many thanks for this suggestion. We have now added these results to the paper.

“Prevalence of disability was higher among those with no education compared to those with education; those not working compared to those in any working category; those not in a union compared with those in a union; and similar among different household possession score groups. These relationships remain when controlling for age and sex”. We have also added the variable "Household Possession Score" to Tables 1 and 2. 

**For the Malawi results using the Malawi Integrated Household Survey 2010/11 and for the Uganda and Ethiopia results on changes in disability status over time, the correct reference is Mitra S: Disability, Health and Human Development. Palgrave MacMillan: New York, and not the article in AJPA.**

Thank you for highlighting this, it has been corrected.

**Regarding the Malawi results using the Malawi Integrated Household Survey 2010/11, the authors of this paper mention the potentially different age structures of the population in the national survey compared to their survey. They should actually check on that given the results in the book above (Table 4.4).**

Thank you. Having revisited the national survey results and compared the age-specific prevalence, we recognise that the age structure of the two populations samples will not explain the difference, so we have removed that statement from our manuscript.  

**The wording of the abstract needs to be carefully edited for accuracy. For instance, the association of obesity and disability is found only for women, but is presented as a general result in the abstract.**

We have updated this wording in the Abstract and also the Conclusion.

**The authors note that “the WG questions do not capture a complete picture of disability, as they do not include pain or low mood and focus more on functional limitations than participation. Our use of this tool therefore meant that we were not able to assess participation restriction, which is a fundamental component of the ICF model of disability. However, the brevity of the WG questions does allow the questions to be easily added to existing surveys, and we did also report on employment inclusion and marital status as proxies for participation”. The WG questions combined with other questions for instance on employment can be used to assess participation restrictions. Hence, this text is unnecessary. It was not within the scope of the paper to assess participation restriction, which is okay.**

 We have amended the wording here and removed some of this explanation.

**Sometimes in the text, LMIC need to be switched to plural.**

We have corrected this, thank you.

Yours sincerely,

Josephine Prynn

# Peer review: Reviewer response for version 1

**DOI:** 10.21956/wellcomeopenres.16579.r36026

**Published:** 2019-08-08

**Authors:** Nawi Ng

**Affiliations:** 1 School of Public Health and Community Medicine Institution of Medicine, Sahlgrenska Academy, University of Gothenburg, Gothenburg, Sweden

Thank you for the opportunity to review this manuscript. I have read the paper with high interest and have comments for the authors to consider in revising their manuscript. 

 The work is not clearly and accurately presented. It is not easy to understand what messages the authors want to deliver. There are quite a lot of analysis results presented in the paper, without relevant rationale. The thread between different parts of the paper is missing. 

 The objectives are not well-phrased, and different concepts seem to be misused.  

- Did the authors want to measure the prevalence or incidence of disability? Or both? The term "incidence" only appears in the title and objective of the paper, but nowhere else. With the follow-up data in the HDSS setting, the authors should be able to estimate the incidence of disability among the study population. The use of panel data would strengthen the quality of the paper. 
- What is the rationale of assessing "consistency" disability reported in two rounds of population census? In research, the term "consistency" is closely related to "reliability". A measure is considered to be reliable if it yields similar results under similar/consistent conditions. The physical, mental and social conditions at different time points can influence the self-reported disability measured 1-2 years apart. Therefore, the two measurements were not done under consistent conditions. Any changes observed in the self-reported disability measured 1-2 years apart could, therefore, reflect real changes in the health conditions, not merely consistency in reporting the disability.  
- What is the rationale of including "overweight and obesity" as a chronic disease?
- Though the authors had access to two rounds of panel data on disability (round 1 in 2014-2015 and round 2 in 2015-2016), this study was designed as a cross-sectional study. The study combined population-based data (NCD survey in 2013-2015, HIV serosurvey in 2011) or hospital data (HIV data from ART clinics) collected on different occasions. There is a serious threat of selection bias due to non-participation in surveys and possibly data missing not in random, which could yield invalid estimates reported in this paper.
- How could the authors ensure that data from different studies were collected using the same protocols? In other word, are the data comparable and is there no threat of misclassification bias? For example, the authors wrote, "Body mass index (BMI) was also taken from the NCD survey unless data on height and weight was available from other studies in the same population obtained closer to the date of the Round 1 census". The authors should be transparent and declare what those other studies are. 
- Though the authors did not attempt to ascertain causality, it is still important to ensure that all the exposure variables were measured before the measurement of disability. Some of the exposure data might be collected after the measurement of disability. 
- As not all the study sample who had disability data in Round 1 (n=10863) participated in the other surveys, information about their BMI, hypertension, diabetes, and HIV status (as shown in Table 1) were therefore missing. Only individuals who had complete information on the exposures and outcome (disability) will be included in the regression analysis when all these variables are included in the model (as shown in Model 1 in Table 3). It is unclear how many these individuals were, as Model 1 might not be valid if it might be based on a small number of individuals.  
- As there are significant amounts of missing data in the dataset, the authors should consider doing multiple imputation. But before coming to this decision, please consider the appropriateness to link data from different sources as discussed above. 
- I do not see the reason to estimate the simulated prevalence of self-reported disability by direct standardisation of the age- and sex-specific disability prevalence in 2014 to the population structure the previous ten years. By doing this, the authors assume that all other factors affecting disability were constant over time. This assumption is invalid. Our previous study on disability in low-and-middle-income countries shows the different factors affecting disability in different countries ^1^.  
- As the age and sex of the respondents who were successfully recruited into Round 1 differ significantly (see 1st paragraph in the Results section), it is important for the authors to weigh all the analyses to get a valid estimate of disability in the population. Otherwise, I suspect that the prevalence of self-reported disability in this study is over-estimated. This is mainly due to the larger number of older population and women who participated in the study. 

The following are additional issues that the author should address to improve the clarity of the text. 

- The authors need to give more details on how the composite score of socioeconomic status was calculated. What statistical method was used to do the analysis? 
- It is surprising to see that there are significant missing data in the socioeconomic variables collected in an HDSS setting. One would expect to see reasonably good quality household-level data from a well-functioning HDSS. 
- Table 1 and 2: The total columns are not needed. As there are many differences between sexes, it is more appropriate to present sex-stratified analyses. 
- Table 3 needs to be revised to make it more comprehensible. Please indicate if Model 1 is a multivariable model, and not bivariate models of all the variables. If hypertension and diabetes were included in the analysis in Model 2, why weren't their results presented in the table? Same question for BMI in Model 3. 
- Table 4. 9.1% respondents could not walk at all in the baseline and reported no difficulty in the follow-up. How could this be explained considering the research setting?  

In brief, I would suggest the authors reformulate the objectives of this study, assess what data sources could be used to address the research questions (considering the limitation of linking the different datasets), weight all the analyses and conduct multiple imputation in order to get valid estimates to be reported in this study. 

 I hope my comments are not too harsh. I wish the authors all the best in revising the work.

Is the work clearly and accurately presented and does it cite the current literature?

Partly

If applicable, is the statistical analysis and its interpretation appropriate?

No

Are all the source data underlying the results available to ensure full reproducibility?

No

Is the study design appropriate and is the work technically sound?

Partly

Are the conclusions drawn adequately supported by the results?

Partly

Are sufficient details of methods and analysis provided to allow replication by others?

No

Reviewer Expertise:

Ageing and disability, risk factor surveillance, epidemiology analysis, longitudinal data analysis.

I confirm that I have read this submission and believe that I have an appropriate level of expertise to state that I do not consider it to be of an acceptable scientific standard, for reasons outlined above.

## References

[1] : Diversity in the Factors Associated with ADL-Related Disability among Older People in Six Middle-Income Countries: A Cross-Country Comparison. *International Journal of Environmental Research and Public Health*.2019;16(8) : 10.3390/ijerph1608134110.3390/ijerph16081341PMC651827631013975

# Response

**Published:** 2019-11-18

**Authors:** Josephine Prynn

**Affiliations:** University College London, UK

Dear Professor Ng,

Many thanks for your considered comments. We have made some major changes to the submission based on these and believe we have improved its quality. We have clarified our findings on incidence of disability, dropped the simulation of disability prevalence over time, and reviewed the way we were considering the panel data and changes in responses between the two rounds. We hope that you will find that this updated version meets your approval.

Detailed responses to your comments below:

Did the authors want to measure the prevalence or incidence of disability? Or both? The term "incidence" only appears in the title and objective of the paper, but nowhere else. With the follow-up data in the HDSS setting, the authors should be able to estimate the incidence of disability among the study population. The use of panel data would strengthen the quality of the paper.

**Response: We have updated Table 4 to include disability incidence over the year between the two rounds.**

What is the rationale of assessing "consistency" disability reported in two rounds of population census? In research, the term "consistency" is closely related to "reliability". A measure is considered to be reliable if it yields similar results under similar/consistent conditions. The physical, mental and social conditions at different time points can influence the self-reported disability measured 1-2 years apart. Therefore, the two measurements were not done under consistent conditions. Any changes observed in the self-reported disability measured 1-2 years apart could, therefore, reflect real changes in the health conditions, not merely consistency in reporting the disability. 

**Response: Thank you for this point. We have changed the wording from consistency to simply describing changes between the two rounds. We discuss how these changes may represent real changes in health conditions, or differences in reporting over time.**

What is the rationale of including "overweight and obesity" as a chronic disease?

**Response: Obesity and overweight are well-recognised risk factors for multiple health conditions and as such was of interest to us. We have changed the wording from chronic diseases to chronic conditions however, as we recognise that obesity itself may not be considered a disease.**

Though the authors had access to two rounds of panel data on disability (round 1 in 2014-2015 and round 2 in 2015-2016), this study was designed as a cross-sectional study. The study combined population-based data (NCD survey in 2013-2015, HIV serosurvey in 2011) or hospital data (HIV data from ART clinics) collected on different occasions. There is a serious threat of selection bias due to non-participation in surveys and possibly data missing not in random, which could yield invalid estimates reported in this paper.

**Response:  Use of all available data sources on HIV was important to minimise the amount of missing HIV data. We have acknowledged the risk of bias from missing data on the different health states and have now added a description of the pattern of missing data into the results to further inform the reader.**

How could the authors ensure that data from different studies were collected using the same protocols? In other word, are the data comparable and is there no threat of misclassification bias? For example, the authors wrote, "Body mass index (BMI) was also taken from the NCD survey unless data on height and weight was available from other studies in the same population obtained closer to the date of the Round 1 census". The authors should be transparent and declare what those other studies are.

**Response:** **We have clarified that only 0.8% of BMI data came from studies other than the NCD survey or the census. The NCD survey, the census, and all of the other 9 studies were performed by the Malawi Epidemiology and Intervention Research Unit using an identical protocol, and in many cases the same members of staff were performing the measurements.**

Though the authors did not attempt to ascertain causality, it is still important to ensure that all the exposure variables were measured before the measurement of disability. Some of the exposure data might be collected after the measurement of disability. Some of the BMI data was collected after the measurement of disability.

**Response:  A sensitivity analysis has been added excluding any BMI data collected after the measurement of disability.**

As not all the study sample who had disability data in Round 1 (n=10863) participated in the other surveys, information about their BMI, hypertension, diabetes, and HIV status (as shown in Table 1) were therefore missing. Only individuals who had complete information on the exposures and outcome (disability) will be included in the regression analysis when all these variables are included in the model (as shown in Model 1 in Table 3). It is unclear how many these individuals were, as Model 1 might not be valid if it might be based on a small number of individuals.  As there are significant amounts of missing data in the dataset, the authors should consider doing multiple imputation. But before coming to this decision, please consider the appropriateness to link data from different sources as discussed above.

**Response:  To reduce the amount of data excluded from the regression models, we have now included an “unknown” category when the variable is a covariate rather than the exposure of interest.**

I do not see the reason to estimate the simulated prevalence of self-reported disability by direct standardisation of the age- and sex-specific disability prevalence in 2014 to the population structure the previous ten years. By doing this, the authors assume that all other factors affecting disability were constant over time. This assumption is invalid. Our previous study on disability in low-and-middle-income countries shows the different factors affecting disability in different countries1.  

**Response:  We have removed this analysis from the paper.**

As the age and sex of the respondents who were successfully recruited into Round 1 differ significantly (see 1st paragraph in the Results section), it is important for the authors to weigh all the analyses to get a valid estimate of disability in the population. Otherwise, I suspect that the prevalence of self-reported disability in this study is over-estimated. This is mainly due to the larger number of older population and women who participated in the study.

**Response:  We now also include age-specific prevalence weighted to the age-population of the underlying census population.**

The following are additional issues that the author should address to improve the clarity of the text.

The authors need to give more details on how the composite score of socioeconomic status was calculated. What statistical method was used to do the analysis?

**Response:  Thank you for this comment and apologies for the lack of clarity. In fact, we didn't use a composite score of socio-economic status – we have updated the text in the Methods section to clarify that. A household possession score was used in the analysis and considered as a potential confounder, but not included in any of the tables nor the final analysis, so we do not currently provide detailed information on how it was calculated.  The variables occupation and education can be considered as proxies for SES.**

It is surprising to see that there are significant missing data in the socioeconomic variables collected in an HDSS setting. One would expect to see reasonably good quality household-level data from a well-functioning HDSS.

**Response:  Household possession score data was gathered from the NCD survey rather than the HDSS census and had some missing data. The other proxies we considered for socio-economic status were “mobile phone use” and “education”, neither of which had considerable missing data. We have changed the text in the Methods section to clarify that.** 

Table 1 and 2: The total columns are not needed. As there are many differences between sexes, it is more appropriate to present sex-stratified analyses.

**Response:  We have updated the tables to remove the totals columns and present sex-stratified columns.**

Table 3 needs to be revised to make it more comprehensible. Please indicate if Model 1 is a multivariable model, and not bivariate models of all the variables. If hypertension and diabetes were included in the analysis in Model 2, why weren't their results presented in the table? Same question for BMI in Model 3 **.**

**Response:  The text of the Statistical Analysis section of the Methods and the headings and footnotes of the tables have been updated to clarify this. Model 1 is a bivariate model; hypertension and diabetes were included in Model 2, but the exposure of interest in that model is BMI**.

Table 4. 9.1% respondents could not walk at all in the baseline and reported no difficulty in the follow-up. How could this be explained considering the research setting?

**Response:  The percentage of 9.1% you mention represents small numbers (2/22) and has wide confidence intervals. It may represent participants with acute illness that had resolved in the intervening time, or impairments that were successfully treated. This has been added to the Discussion section.**

In brief, I would suggest the authors reformulate the objectives of this study, assess what data sources could be used to address the research questions (considering the limitation of linking the different datasets), weight all the analyses and conduct multiple imputation in order to get valid estimates to be reported in this study.

**Response:  Many thanks for these comments, which have undoubtedly led to an improved quality of this submission. As outlined above, we have changed the objectives of the study to remove the simulation of disability prevalence over time, and better address the question of incidence of disability. We have updated the interpretation of the panel data as you suggested, weighted our prevalence estimates to the underlying census population, and added more detail and sensitivity analyses to address the issue of missing data.**

 Many thanks again for your time and your valuable comments.

# Peer review: Reviewer response for version 1

**DOI:** 10.21956/wellcomeopenres.16579.r35881

**Published:** 2019-07-22

**Authors:** Sophie Mitra

**Affiliations:** 1 Fordham University, New York, NY, USA

The paper deals with a very important and yet neglected topic: disability prevalence and its correlates in a resource poor setting. It is very well written and the analysis is carefully executed. I think the biggest contribution of this paper is to study the prevalence of functional limitations and the health conditions associated with functional limitations in a low-income setting.

 I have several major comments that need to be addressed for the paper to become indexed.

**1. Methods**

- Definition of the category of disability should be expanded to include ‘some difficulty’ at least in a sensitivity analysis.
- The authors strictly follow the recommendations of the WG that focus on only two categories (a lot of difficulty and unable to do) but there is work to figure out how disability analyses change as this definition is expanded to include ‘some difficulty’.
- In addition, the authors find that “most people who reported 'some difficulty' or 'a lot of difficulty' changed category, usually with an improvement in functional”. This is a very important result. In addition, in the discussion, the authors note: “over time between “some difficulty” (categorised as no disability) and “a lot of difficulty” (categorized as having disability), using this cut-off may lead to measurement error and imprecision in estimates of associations and trends.”
- Recently, one study (Mitra 2018 ^1^) also studied people reporting some difficulty under disability, although this is not strictly in line with the recommendations of the WG that focus on only two categories (a lot of difficulty and unabe to do) as done in this study. Given the results above and given similar results in Mitra (2018) ^1^ using panel data on disability for Ethiopia and Uganda using two waves of data, we recommend that the authors include the additional category ‘some difficulty’ in their analysis (or at least in a sensitivity analysis) and mention results of disability prevalence and correlates when the group with some difficulty is included. This affects results in Tables 2 and 3 and Fig 4.
- Figure 3: I do not understand the point of doing the simulation. This should be dropped (or at the very least motivated convincingly).

**2. Motivation, Contribution of the study and review of the literature.**

- The authors should motivate the analysis in light of recent related calls for research on disability and development (UN 2018 ^2^, Groce and Mont 2018 ^3^, Mitra 2018b ^4^).
- The authors need to think more carefully about the contribution of their study in light of a thorough review of the literature on disability in low-income settings. I think that the main contribution of this paper is to study the prevalence of functional limitations and the health conditions associated with functional limitations in a low income setting. To my knowledge, the association with health conditions using biomarkers as done in this study (except for HIV) has not been done before.
- In contrast, the exploitation of longitudinal data on functional limitations has been done before. Under Discussion, the authors write “No studies from LMICs have previously reported on consistency in disability categorisation over time.” Please note that Mitra (2018a) ^1^ does that for two countries in Africa, so the statement should be qualified. To my knowledge, this is true for Malawi.
- The authors should also compare their results with those on Malawi in Mitra (2018a) ^1^.

**3. A number of edits are required throughout the paper to improve clarity. Some are noted below.**

- Abstract/conclusion: “Further research into the needs of this population is crucial”: I suggest replacing “needs” with “situation”.
- Introduction: “The prevailing framework for conceptualising disability is using the World Health Organization (WHO) International Classification of Functioning and Disease ^1^.” Instead of “prevailing” I recommend, “commonly used”.
  Instead of “or more holistically on participation and disability”, I recommend “or more holistically on participation and activities”.
  Also, the authors should note if they use ‘disability’ as an umbrella term for impairments, activity limitations and participation restrictions as in the ICF. It sounds like they do in the introduction. However, later, they use the term as per the Washington Group measure referring to a lot of difficulty or unable to do difficulty in at least one of six domains. If they use it as an umbrella term (which is consistent with the ICF, their conceptual framework), then it would be more precise to simply refer to functional/activity limitations for their empirical results using the WG questions on functional/activity limitations.
- Methods: Please indicate if the six questions of the Washington Group were preceded by an introduction sentence clarifying that the questions are about difficulties related to health.
  The term “self reported difficulty’ is used several times. I think this is unnecessary. It is enough to indicate once under Methods that functional difficulties are self-reported.
- Discussion: The term ‘objective’ to discuss disability measurement may not be clear to all readers: please use something else or clarify. You may want to use ‘clinical assessment”.
  The term ‘incidence’ is mentioned in the title only. Consider removing or use it (after defining it) in the analysis.

Is the work clearly and accurately presented and does it cite the current literature?

Yes

If applicable, is the statistical analysis and its interpretation appropriate?

Yes

Are all the source data underlying the results available to ensure full reproducibility?

Yes

Is the study design appropriate and is the work technically sound?

Partly

Are the conclusions drawn adequately supported by the results?

Partly

Are sufficient details of methods and analysis provided to allow replication by others?

Partly

Reviewer Expertise:

Disability; economics; public health

I confirm that I have read this submission and believe that I have an appropriate level of expertise to confirm that it is of an acceptable scientific standard, however I have significant reservations, as outlined above.

## References

[1] : Disability, Health and Human Development. *SSRN Electronic Journal*.2018; 10.2139/ssrn.309735510.2139/ssrn.3097355

[2] : UN Flagship Report on Disability and Development 2018. *[Accessed 22nd July 2019]*.2018; Reference source

[3] : Counting disability: emerging consensus on the Washington Group questionnaire. *The Lancet Global Health*.2017;5(7) : 10.1016/S2214-109X(17)30207-3e649-e65010.1016/S2214-109X(17)30207-328619216

[4] : From Disability in Resource-Poor Settings to Policy and Research Opportunities in Global Health.*Am J Public Health*.2018;108(9) : 10.2105/AJPH.2018.3045971163-116510.2105/AJPH.2018.30459730089002PMC6085054

# Response

**Published:** 2019-11-18

**Authors:** Josephine Prynn

**Affiliations:** University College London, UK

Dear Professor Mitra,

Many thanks for your thoughtful comments on our paper. We appreciate the importance of thinking about “some difficulty” as well as “a lot of difficulty” when considering disability as defined by the Washington Group questions, and have updated our analyses accordingly. We have dropped the simulation of disability prevalence over time as you suggested, and we have been able to compare our results to those found using the other published literature that you brought to our attention.

Please find a detailed response to each of the issues raised below.

1. Methods

- Definition of the category of disability should be expanded to include ‘some difficulty’ at least in a sensitivity analysis.

**Response: We have now included the prevalence of at least “some difficulty” into Table 2, and described the findings in the text.**

- The authors strictly follow the recommendations of the WG that focus on only two categories (a lot of difficulty and unable to do) but there is work to figure out how disability analyses change as this definition is expanded to include ‘some difficulty’.  In addition, the authors find that “most people who reported 'some difficulty' or 'a lot of difficulty' changed category, usually with an improvement in functional”. This is a very important result. In addition, in the discussion, the authors note: “over time between “some difficulty” (categorised as no disability) and “a lot of difficulty” (categorized as having disability), using this cut-off may lead to measurement error and imprecision in estimates of associations and trends.” Recently, one study (Mitra 20181) also studied people reporting some difficulty under disability, although this is not strictly in line with the recommendations of the WG that focus on only two categories (a lot of difficulty and unabe to do) as done in this study. Given the results above and given similar results in Mitra (2018)1 using panel data on disability for Ethiopia and Uganda using two waves of data, we recommend that the authors include the additional category ‘some difficulty’ in their analysis (or at least in a sensitivity analysis) and mention results of disability prevalence and correlates when the group with some difficulty is included. This affects results in Tables 2 and 3 and Fig 4.

**Response**: **We have added a sensitivity analysis using at least “some difficulty” as an outcome, available in the Extended Data.** 

- Figure 3: I do not understand the point of doing the simulation. This should be dropped (or at the very least motivated convincingly).

**Response: We have dropped this.**

2. Motivation, Contribution of the study and review of the literature.

- The authors should motivate the analysis in light of recent related calls for research on disability and development (UN 20182, Groce and Mont 20183, Mitra 2018b4).

**Response: We have strengthened our motivation in the Introduction.**

- The authors need to think more carefully about the contribution of their study in light of a thorough review of the literature on disability in low-income settings. I think that the main contribution of this paper is to study the prevalence of functional limitations and the health conditions associated with functional limitations in a low income setting. To my knowledge, the association with health conditions using biomarkers as done in this study (except for HIV) has not been done before.

**Response**: **We have changed the wording in the Discussion and Conclusion to highlight this.**

- In contrast, the exploitation of longitudinal data on functional limitations has been done before. Under Discussion, the authors write “No studies from LMICs have previously reported on consistency in disability categorisation over time.” Please note that Mitra (2018a)1 does that for two countries in Africa, so the statement should be qualified. To my knowledge, this is true for Malawi.

**Response**: **We have changed wording and now also compare results to those in Mitra 2018 for Ethiopia and Uganda.**

- The authors should also compare their results with those on Malawi in Mitra (2018a)1.

**Response**: **This comparison is now included.**

3. A number of edits are required throughout the paper to improve clarity. Some are noted below.

- Abstract/conclusion: “Further research into the needs of this population is crucial”: I suggest replacing “needs” with “situation”.

**Response**: **Done.**

- Introduction: “The prevailing framework for conceptualising disability is using the World Health Organization (WHO) International Classification of Functioning and Disease1.” Instead of “prevailing” I recommend, “commonly used”.

**Response**: **Done.**

- Instead of “or more holistically on participation and disability”, I recommend “or more holistically on participation and activities”.

**Response**: **Done.**

- Also, the authors should note if they use ‘disability’ as an umbrella term for impairments, activity limitations and participation restrictions as in the ICF. It sounds like they do in the introduction. However, later, they use the term as per the Washington Group measure referring to a lot of difficulty or unable to do difficulty in at least one of six domains. If they use it as an umbrella term (which is consistent with the ICF, their conceptual framework), then it would be more precise to simply refer to functional/activity limitations for their empirical results using the WG questions on functional/activity limitations.

**Response**: **Thank you for your comment on this. Our use of the Washington Group questions meant that we were unable to assess participation restriction, which we acknowledge is a fundamental component of the ICF model of disability, and have added an acknowledgement and explanation of this in the Discussion.   ** 

- Methods: Please indicate if the six questions of the Washington Group were preceded by an introduction sentence clarifying that the questions are about difficulties related to health. 

**Response**: **The questions are asked within a section of questions related to health and fertility. We have added this information to the Methods section. **

- The term “self reported difficulty’ is used several times. I think this is unnecessary. It is enough to indicate once under Methods that functional difficulties are self-reported.

**Response**: **Thanks for this comment – we have updated the prose accordingly. **

- Discussion: The term ‘objective’ to discuss disability measurement may not be clear to all readers: please use something else or clarify. You may want to use ‘clinical assessment”.

**Response**: **Done.**

- The term ‘incidence’ is mentioned in the title only. Consider removing or use it (after defining it) in the analysis.

**Response**: **We have now included incidence in Table 4 using the panel data from the 2 consecutive surveys.**

Many thanks again for your time.
